# Indirect effects of invasive rat removal result in recovery of island rocky intertidal community structure

**DOI:** 10.1038/s41598-021-84342-2

**Published:** 2021-03-08

**Authors:** Carolyn M. Kurle, Kelly M. Zilliacus, Jenna Sparks, Jen Curl, Mila Bock, Stacey Buckelew, Jeffrey C. Williams, Coral A. Wolf, Nick D. Holmes, Jonathan Plissner, Gregg R. Howald, Bernie R. Tershy, Donald A. Croll

**Affiliations:** 1 Division of Biological Sciences, Ecology, Behavior, and Evolution Section, University of California San Diego, 9500 Gilman Dr, La Jolla, CA 92023 USA; 2 Conservation Action Lab, University of California Santa Cruz, 115 McAllister Way, Santa Cruz, CA 95060 USA; 3 Island Conservation, 2100 Delaware Ave, Suite 1, Santa Cruz, CA 95060 USA; 4 US Fish and Wildlife Service, Alaska Maritime National Wildlife Refuge, 95 Sterling Highway, Suite 1, Homer, AK 99603 USA; 5 Present Address: Oikonos Ecosystem Knowledge, PO Box 2570, Santa Cruz, CA 95063 USA; 6 Present Address: Alaska Department of Fish and Game, Division of Wildlife Conservation, 1300 College Rd, Fairbanks, AK 99701 USA; 7 Present Address: Great Basin Institute, 16750 Mt. Rose Highway, Reno, NV 89511 USA; 8 Present Address: Axiom Data Science, 1016 W 6th Ave, Ste. 105, Anchorage, AK 99501 USA; 9 Present Address: The Nature Conservancy, 201 Mission Street #4, San Francisco, CA 94105 USA; 10 Present Address: FreshWater Life, Telluride, CO USA

**Keywords:** Conservation biology, Community ecology, Food webs, Ecology, Conservation biology

## Abstract

Eleven years after invasive Norway rats (*Rattus norvegicus*) were eradicated from Hawadax Island, in the Aleutian Islands, Alaska, the predicted three-level trophic cascade in the rocky intertidal, with native shorebirds as the apex predator, returned, leading to a community resembling those on rat-free islands with significant decreases in invertebrate species abundances and increases in fleshy algal cover. Rats had indirectly structured the intertidal community via their role as the apex predator in a four-level trophic cascade. Our results are an excellent example of an achievable and relatively short-term community-level recovery following removal of invasive animals. These conservation successes are especially important for islands as their disproportionately high levels of native biodiversity are excessively threatened by invasive mammals.

## Introduction

Invasive animals are a main driver of global biodiversity loss and can impact ecosystem function^1–5^. Invasive animal impacts are particularly disruptive on islands because islands usually have small numbers of species resulting in simplified food chains and limited functional redundancy, and those species typically have limited evolved defenses against herbivory, predation, and competition^6^. The direct effects of invasive animals on islands are well documented^7–11^, and there is increasing evidence of their multiple indirect effects as well^6, 12–15^.

Invasive rats (*Rattus spp.*) are the most widespread and damaging invasive animals^16,17^. They are present on perhaps 90% of islands^18,19^, influence island biota both directly as competitors, predators, herbivores, and frugivores^19–21^, and indirectly via trophic cascades, cross ecosystem subsidies, propagule dispersal, and mutualist networks^9,13,14,22–24^.

Because of these deleterious impacts, the removal of invasive animals has become an important global conservation strategy, with over 900 successful animal eradications on almost 800 islands since 1950^25^. While the number, rate, and size of invasive animal eradications is increasing^8, 26–28^, studies of the full benefits to island communities after invasive animal eradications are limited (but see^8^). Longer-term post-eradication studies of island community recoveries from the direct and indirect impacts of invaders are even more limited^29^, as are recoveries from the effects of cross-ecosystem (e.g. near-shore marine vs. terrestrial) interactions.

Community recovery after invasive animal eradication is difficult to measure for many reasons, including natural stochasticity and uncertain baselines by which to compare altered landscapes. In addition, demonstration of community recovery requires study of the indirect effects of invaders, adequate measures of multiple community components, and a commitment to long-term monitoring. Therefore, the extent to which entire communities recover following invasive species eradication, and the time required for recovery, are much less understood than the direct deleterious effects of invaders and the recoveries of multiple native species once invaders and their direct mechanisms of control (frequently predation) are removed (but see^30,31^).

**Figure 1:**
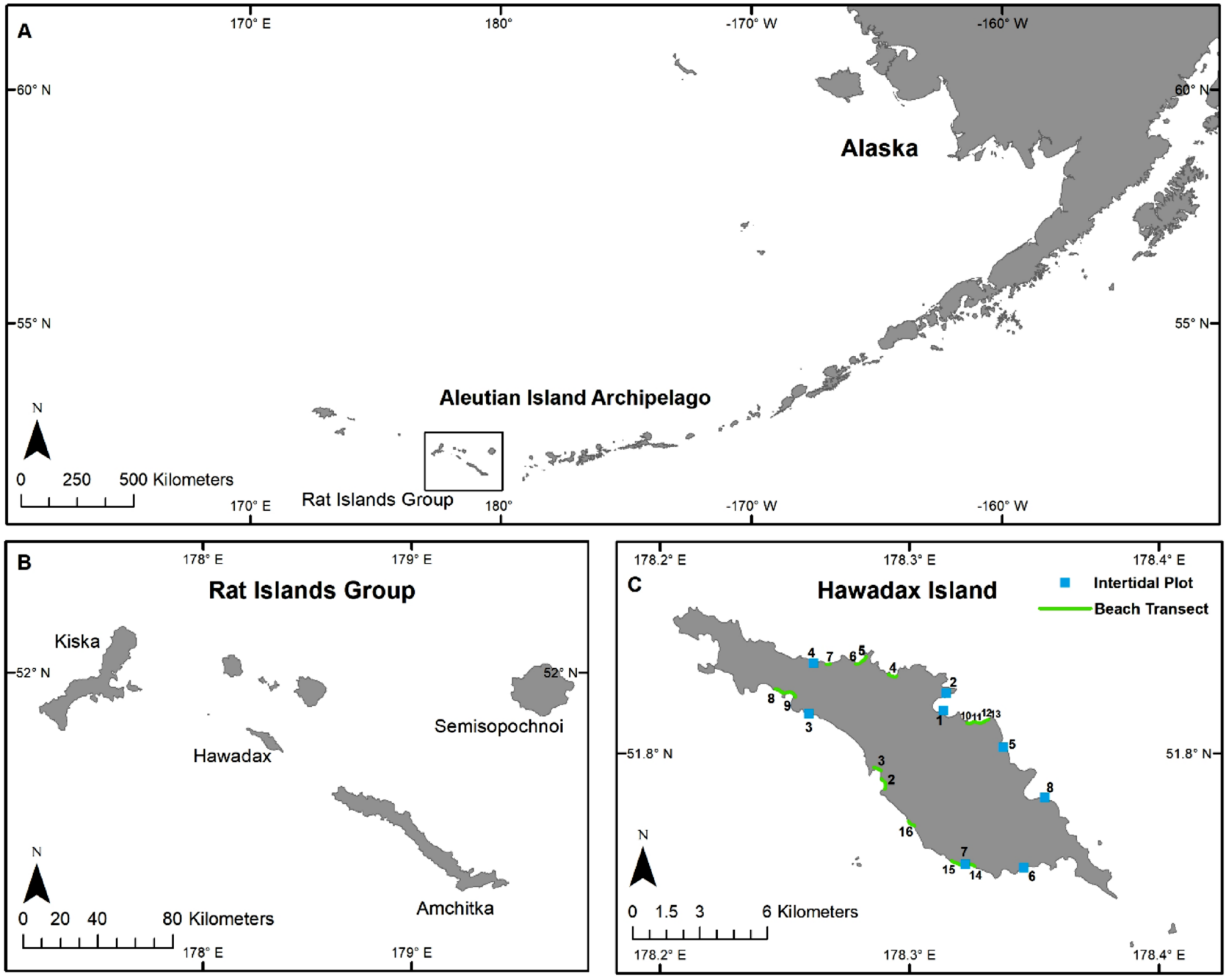
The (**A**) central Aleutian Archipelago, Alaska, USA contains the (**B**) Rat Islands Group, including Hawadax Island. The (**C**) intertidal plots and beach transects surveyed on Hawadax Island. Intertidal plot 6 was only surveyed in 2008 and 2013^35^.

Hawadax Island (previously known as Rat Island) is in the central Aleutian Island chain (Fig. 1). The island was likely invaded by Norway rats (*R. norvegicus*) when a Japanese ship went aground in the 1780′s, and invaded by Arctic foxes (*Vulpes lagopus*) following intentional introductions by fur traders in the 1800s^32^. The combined impact of an introduced carnivore (foxes) and omnivore (rats) had multiple direct and indirect impacts ^33^. For example, while archeological evidence from native Unangan (Aleut) habitation sites indicate marine birds were once common on Hawadax Island, rats and introduced foxes extirpated locally breeding seabirds, shorebirds, and land birds^32^.

Foxes were eradicated from Hawadax Island in 1984^34^, and rats were eradicated in 2008 using aerially broadcast rodenticide (25-ppm brodifacoum^32^). Croll et al.^33^ reported on the direct effects of rat eradication after five years, demonstrating significant recoveries of terrestrial birds (Gray-crowned Rosy Finch [*Leucosticte tephrocotis*], Lapland Longspur [*Calcarius lapponi-cus*], Snow Bunting [*Plectrophenax nivalis*], and Song Sparrow [*Melospiza melodia*]) and shorebirds (Black Oystercatcher [*Haematopus bachmani*] and Rock Sandpiper [*Calidris ptilocnemis*], and the initial recolonization or recovery of marine birds (Tufted Puffin [*Fratercula cirrhata*], Leach’s Storm-petrel [*Oceanodroma leucohoa*], ﻿and Glaucous-winged Gull [*Larus glaucescens*]). However, community-level changes resulting from indirect changes in trophic structure post-rat eradication are likely to take longer than the recovery of these directly impacted bird species^30^.

**Figure 2:**
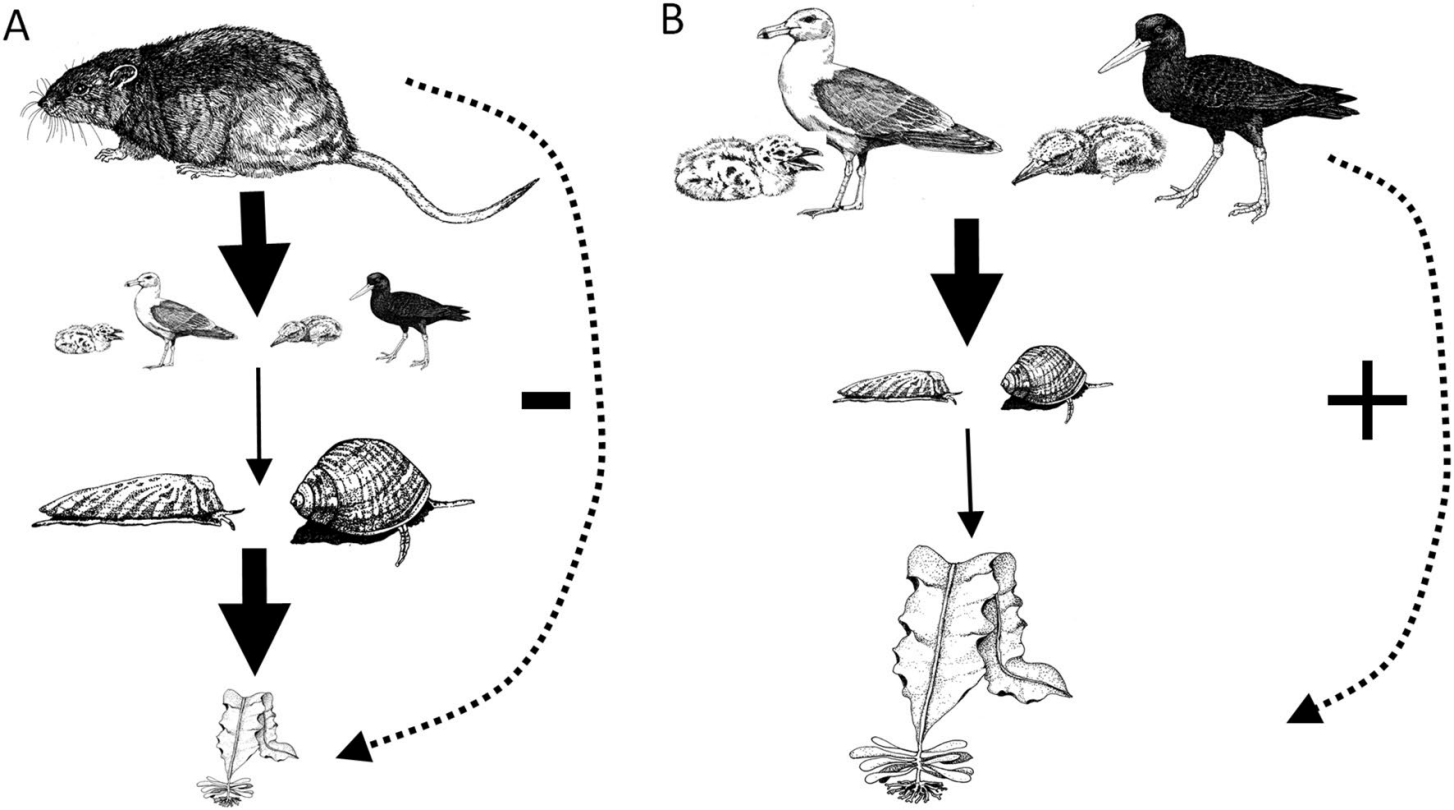
The presence of invasive rats on Aleutian Islands in Alaska creates a (**A**) four-level trophic cascade wherein rats negatively impact primary productivity, indirectly (dotted line) turning the rocky intertidal community into an invertebrate dominated system by depredating shorebirds and releasing intertidal grazers from bird predation pressure. On islands without rats, and presumably on islands in recovery after rat removal, such as Hawadax, the rocky intertidal becomes a (**B**) three-level trophic cascade wherein shorebirds depredate herbivorous invertebrates, thereby releasing algae from grazing pressure, and indirectly creating an algal dominated community. Birds also consume invertebrate non-grazers (e.g., mussels, anemones, seastars, and sponges), and their decreased abundances following rat removal may lead to increased availability of space in the rocky intertidal, further facilitating increases in algal cover. Figure modified from Kurle et al.^12.^, Gena Bentall drew the images and C. Kurle created the figure.

To measure the time required and potential for passive community recovery on an island after rat eradication, we measured abundances of shorebirds and multiple rocky intertidal species before (2008), and five (2013) and 11 (2019) years post-rat eradication on Hawadax Island. Previous comparisons in 2002–2004 by Kurle et al.^12^ of the rocky intertidal community composition on Islands in the Aleutian archipelago with and without invasive rats demonstrated a rat-mediated, four-level trophic cascade in which rats extirpated the shorebirds that forage on intertidal herbivores, leaving a system dominated by invertebrate herbivores (Fig. 2). Kurle et al.^12^ found that 83% and 96% of islands could be assigned to their correct category of rat-infested or rat-free, respectively, based on the species composition of the rocky intertidal. Thus, we predicted that the rocky intertidal community on Hawadax Island would return to a state resembling a rat-free island given sufficient time for shorebird populations to recover to levels necessary to maintain the three-trophic level cascade typical of Aleutian Islands without rats.

We compared our long-term monitoring data collected on Hawadax Island pre- (2008) and post- (2013 and 2019) rat eradication to data contrasting rat-infested and rat-free Aleutian Islands measured in 2002–2004 from Kurle et al.^12^. Specifically, we sought to: (1) document longer-term recovery of breeding intertidal-feeding marine birds (Black Oystercatchers and Glaucous-winged Gulls), (2) examine changes in the marine rocky intertidal community related to the direct and indirect impacts of marine bird recovery, and (3) better understand the time required for recovery of marine rocky intertidal communities on islands after rat removal.

## Results

### Intertidal community

**Figure 3:**
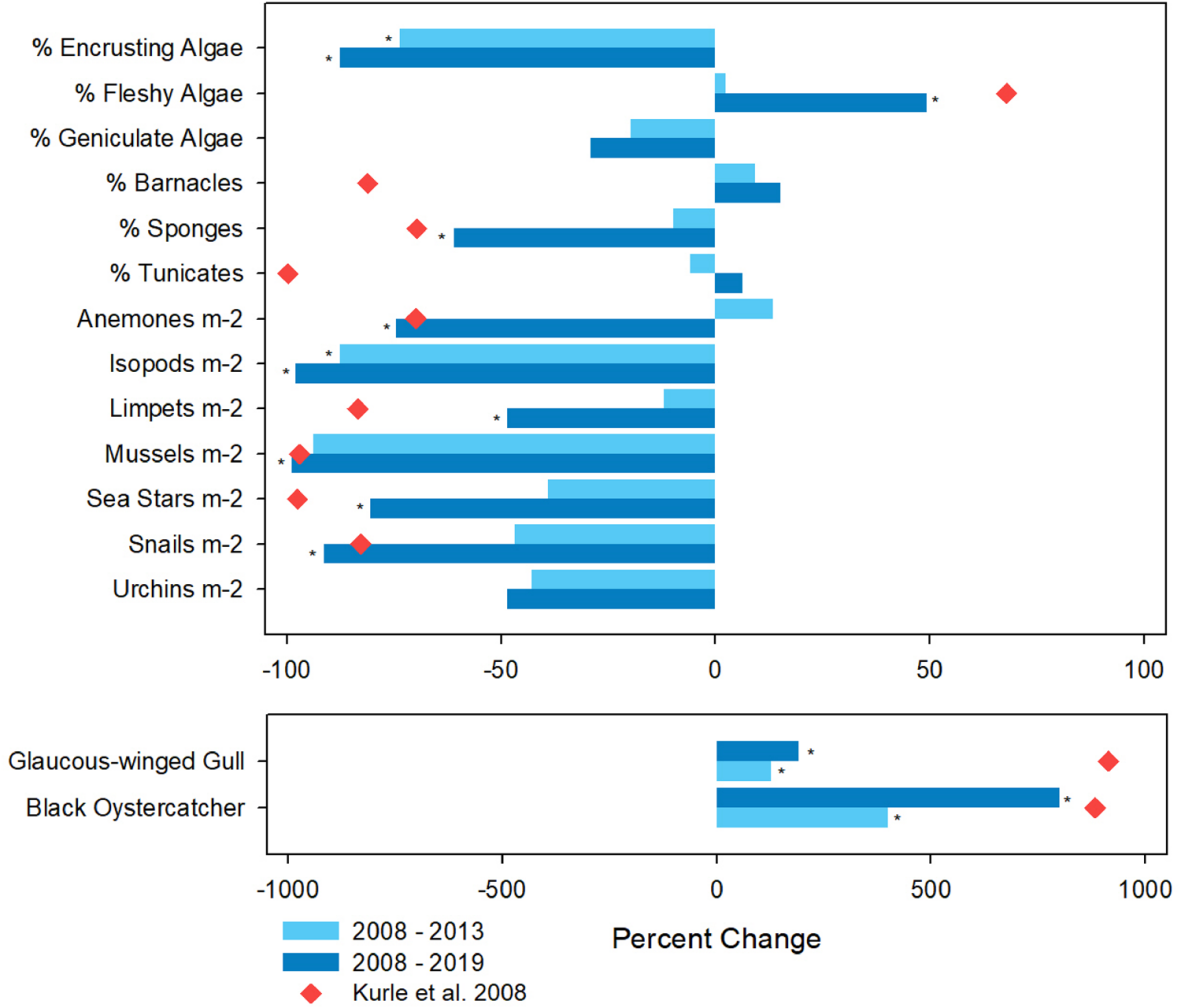
The percent change in algal, barnacle, sponge, and tunicate percent cover and mean number per m^2^ of invertebrates pre- vs. post-eradication (2008 vs. 2013 and 2019) measured from intertidal photo quadrats. * indicates significantly different data between 2008 and 2013 or 2008 and 2019, *p* < 0.05. The red diamonds indicate the percent change between islands with rats and without rats from Kurle et al.^12^. No red diamond indicates an organism that was not measured in Kurle et al.^12^.

Predicted changes in the rocky intertidal community^12^ were largely not evident five years post-rat eradication (Table 1, Fig. 3, and Supplementary Fig. 1). However, by 11 years post-eradication, seven of 13 intertidal taxonomic groups showed significant predicted changes (Table 1, Fig. 3, Supplementary Fig. 1). Three species (anemones, mussels, and snails) met or exceeded the expected percent changes in abundance over time observed between islands with and without rats. One (sponges) was within less than 10% of the expected abundance

**Table 1:** The relative abundance of intertidal organisms measured before and after rat eradication (Mean ± SE) (2008 and 2013: n = 8; 2019: n = 7).

Intertidal organism	Pre	Post	2008 vs. 2013		Post	2008 vs. 2019	
	2008	2013	t	*P*	2019	t	*P*
% Encrusting Algae	47.9 ± 3.1	12.67 ± 2.84	− 8.3931	< 0.0001*	5.9 ± 1.3	− 12.435	< 0.0001*
% Fleshy Algae	25.5 ± 2.3	26.09 ± 2.36	0.1858	NS	38.0 ± 3.2	3.151	0.0045*
% Geniculate Algae	4.52 ± 1.4	3.62 ± 0.98	− 0.5258	NA	3.2 ± 1.02	− 0.763	0.2298
% Barnacles	0.19 ± 0.11	0.21 ± 0.14	0.0965	NS	0.22 ± 0.18	0.136	0.4472
% Sponges	9.44 ± 2.4	8.51 ± 1.18	− 0.3427	NS	3.69 ± 0.84	− 2.245	0.0263*
% Tunicates	1.99 ± 0.5	1.87 ± 0.59	− 0.1513	NS	2.12 ± 0.69	0.148	0.4424
Anemones m^−2^	66.6 ± 20.9	75.6 ± 33.98	− 0.1141	NS	17.1 ± 10.9	− 3.003	0.0061*
Isopods m^−2^	85.7 ± 20.9	10.68 ± 2.82	− 4.973	0.0001*	1.69 ± 0.63	− 9.195	< 0.0001*
Limpets m^−2^	26.4 ± 4.6	23.19 ± 4.95	− 0.6545	NS	13.5 ± 2.9	− 2.219	0.0255*
Mussels m^−2^	54.9 ± 39.5	3.43 ± 3.15	− 1.5288	NS	0.61 ± 0.47	− 1.934	0.0447*
Sea Stars m^−2^	8.1 ± 3.08	4.95 ± 1.41	− 0.2225	NS	1.59 ± 0.72	− 1.919	0.0395*
Snails m^−2^	359.7 ± 72.1	191.04 ± 45.16	− 1.6452	NS	31.0 ± 11.5	− 5.452	< 0.0001*
Urchins m^−2^	20.3 ± 5.5	11.63 ± 4.33	− 1.2599	NS	10.4 ± 2.34	− 1.088	0.1481

*P is significant with α < 0.05.

change and three (fleshy algae, limpets, and sea stars) were within 17 to 35%. The abundances of two (barnacles and tunicates) did not change as predicted, and the remaining four taxa were not measured in Kurle et al.^12^ (Fig. 3, Supplementary Table 1), so were not compared.

### Beach transects

Glaucous-winged Gulls and Black Oystercatchers were significantly more abundant post-eradication in 2013 and even more so in 2019 (Table 2, Fig. 3, Supplementary Table 1, Supplementary Fig. 1). Kurle et al.^12^ observed that Black Oystercatcher and Glaucus-winged Gull abundances were greater by 984% (0.246 ± SD 0.44 vs. 0.025 ± SD 0.04 per km of shoreline) and 1014% (17.643 ± SD 60.89 vs. 1.740 ± SD 3.09 per km of shoreline), respectively, on islands without rats, whereas we observed 900% and 291% increases in their abundances between 2008 and 2019 (Table 2). In addition, we detected 19 active Glaucous-winged Gull nests and five active Black Oystercatcher nests post-eradication in 2019 compared to five nests and one nest, respectively, pre-eradication in 2008.

**Table 2:** The mean number km^−1^ ± SE of Black Oystercatchers and Glaucous-winged Gulls detected on beach transects “Pre” (2008; n = 16) and “Post” rat eradication (2013; n = 16 and 2019; n = 16).

Species	Pre	Post	2008 vs. 2013		Post	2008 vs. 2019	
	2008	2013	Chi Square	*P*	2019	Chi Square	*P*
Black Oystercatcher	0.001 ± 0.001	0.005 ± 0.001	15.7081	< 0.0001*	0.009 ± 0.002	17.8115	< 0.0001*
Glaucous-winged Gull	0.023 ± 0.008	0.052 ± 0.01	17.0103	< 0.0001*	0.067 ± 0.025	6.1029	0.035*

*P is significant with α < 0.05.

## Discussion

Multiple studies document significant restoration of plant and animal species on islands following invasive animal removal^5,8, 36–39^. In particular, positive growth in bird abundances post-eradication is especially well-documented^31, 40,41^, including in the Aleutian Islands, where bird abundances increased significantly 5–10 years after invasive fox and/or rat eradication^33, 42–44^. However, most of these studies focus on the reestablishment of individual native vertebrate or plant species. It is more difficult to assess the long-term responses of entire communities or ecosystems, whose recoveries are frequently tied to the return of native species known to structure communities via their foraging patterns and other activities (but see^4,45^).

Based on previous work by Kurle et al.^12^ comparing rocky intertidal communities on islands with and without rats in the Aleutian archipelago, we hypothesized that rat removal would eventually return the marine rocky intertidal community on Hawadax Island to a three trophic level system with shorebirds as apex predators, instead of a four trophic level system with rats as apex predators, and thus change from algae- to invertebrate-dominated (Fig. 2). Consistent with this hypothesis, we found a dramatic shift in invertebrate and algal cover dominating the rocky intertidal community on Hawadax Island after rat eradication. Specifically, 11 years post rat eradication, we found: 1) a significant increase in percent cover of fleshy algae, 2) significant decreases in grazers of fleshy algae (isopods, limpets, and snails), as well as four other invertebrate groups (anemones, mussels, seastars, and sponges), and 3) significant increases in the shorebird predators (Glaucous-winged Gulls and Black Oystercatchers) of these intertidal invertebrates both five and 11 years post-rat eradication. Isopods were the only invertebrate that showed a statistically significant decrease in abundance five years post-rat eradication.

In rocky intertidal communities, marine birds can control abundances of invertebrate grazers via predation^46,47^, and intertidal invertebrate herbivores reduce algal cover through grazing pressure^48,49^, leading, in some cases, to three level trophic cascades^50,51^ such as those observed on rat-free islands in Kurle et al.^12^. We expect other processes such as upwelling, temperature, recruitment, and currents also influenced the rocky intertidal community structure across the Aleutian Islands^48, 52^. However, given the many examples of top-down control in rocky intertidal systems (see above), coupled with the patterns Kurle et al.^12^ observed across 23 islands spanning nearly the entire Aleutian Island chain, we are confident that the likely mechanism driving the rocky intertidal community structure on Hawadax Island is the extirpation of rats followed by the recovery of gulls and oystercatchers as the apex predators in a three level trophic cascade.

Abundances of gulls and oystercatchers were significantly greater in 2013 (2.3 and 5 times higher, respectively) compared with 2008, indicating passive recovery of the shorebird populations had already begun five years post rat-eradication. However, the intertidal data suggest this level or time for recovery was not sufficient to restore the rocky intertidal food web to a shorebird-mediated state more resembling that of a rat-free island. By 2019, gull and oystercatcher abundances had further increased to 2.9 and 9 times higher, respectively, than from 2008. These bird numbers are still lower than earlier measures from Aleutian Islands with no history of rat or fox invasions^12^ (n = 89 islands, oystercatchers = 0.25 ± SD 0.44 and gulls = 17.64 ± SD 60.88 birds per km of shoreline). However, the bird numbers between this study and Kurle et al.^12^ are not directly comparable as they derived their estimates from bird counts conducted by personnel circumnavigating islands in small boats rather than the land-based beach transect counts used here.

A few rocky intertidal species measured in this study did not follow the patterns observed in Kurle et al.^12^ between islands with and without rats. First, the percent cover of barnacles and tunicates were not different over time in this study, but their abundances were significantly less on islands without rats than on islands with rats in Kurle et al.^12^. These exceptions may simply reflect dietary choices by gulls and oystercatchers as they do not appear to eat tunicates, and barnacles make up only a small percentage of their preferred intertidal prey^53–56^. Kurle et al.^12^ surmised that the greater area covered by invertebrates not eaten by shorebirds on islands with rats was due to less algal cover and the resultant increased rocky intertidal substrate space available for invertebrate colonization. If this is the case, it may take more time for intertidal community differences related to competition, succession, and space availability to become measurable. In addition, the percent cover of geniculate algae in the intertidal showed no difference between 2008 and 2019. Coralline algal species in the North Pacific are fairly slow to colonize newly opened space^57^ and are initially outcompeted by fleshy algal species and certain invertebrates^58^, which could explain why their abundances did not change over the course of this study.

Further, we found no significant differences in the abundances of sea urchins, a known diet component for shorebirds, between pre- and post-eradication on Hawadax Island. Urchin abundance was not assessed in Kurle et al.^12^. Urchins are difficult to accurately measure in intertidal surveys as they are largely subtidal organisms, remaining submerged throughout a tide cycle by following the tidal flux or via confinement to shallow tide pools^59^. In addition, around the Aleutian Islands, urchin numbers are largely regulated by sea otter (*Enhydra lutris*) predaton. Sea otters around the Aleutian Islands have remained in steep decline since the 1990′s, likely from predation by killer whales (*Orcinus orca*)^60^, and are thus reduced across much of the Archipelago. Therefore, sea urchin abundances are high in many of the subtidal zones around the Aleutian Islands, including around Hawadax^61,62^, and likely not controlled by bird predation.

A large marine heatwave (“The Blob”) began in the Gulf of Alaska in fall 2013, spread south to Baja California, and caused warm sea surface temperature anomalies in the top ~ 100 m of the ocean until April 2015^63^. Long-term monitoring of sites in the Gulf of Alaska (GOA) and the eastern Alaska Peninsula (EAP) documented significant intertidal changes related to The Blob, including decreases in sea stars, increases in mussels, and decreases in fleshy algae^64^. Coletti et al.^64^ related the reduction in sea stars to Sea Star Wasting Disease (SSWD), a syndrome causing mass mortality of sea stars from south-central Alaska to Baja California over the last decade. Mussel densities in the GOA and EAP then rose in response to a reduction in their sea star predators. The decreases in fleshy algal cover in the GOA and EAP were attributed to reductions in survival and/or recruitment of the brown algae *Fucus distichus* related to warmer than normal temperatures^65^. The range of SSWD detected in sea stars does not include the Aleutian Islands^66^, so is unlikely a factor in the decrease in sea stars or mussels we observed. And the trend of decreasing fleshy algal cover related to The Blob is the opposite of the increases we observed, making it unlikely its effects contributed to algal cover changes we observed on Hawadax.

Determining ecological community recovery following restoration is challenging but is aided by the clear definition of a goal, a quantified description of that state (e.g. equivalent monitoring data from experimental or natural controls), and an understanding of other environmental drivers that influence a restoration outcome. In addition, while species compositions are fairly uniform within intertidal communities across the Aleutian Islands, each island is subject to some variation in recruitment, reproduction, competition, predation, wave-action, and other factors influencing the densities and cover of intertidal species, further complicating our ability to assess the degree of intertidal community recovery 11 years after rat eradication on Hawadax Island. However, comparisons of the percentage change over time for the densities and percent cover values of intertidal organisms on Hawadax pre- vs. post-eradication are similar to those observed between islands with and without rats surveyed in 2002–2004 for Kurle et al.^12^ (Fig. 3, Supplementary Table 1), indicating a high degree of recovery from rat impacts in the rocky intertidal.

Across 60 + years of invasion ecology as a discipline^67,68^, research has accumulated overwhelming evidence detailing the loss of biodiversity and other threats to native species and ecosystems posed by non-native invaders^69^, especially on islands^3^. When mammalian invaders are removed from islands, conservation success, if measured at all, is generally tracked by how well populations of native (and largely terrestrial) species rebound^8^. However, less understood or even studied, are recoveries of entire communities, particularly nearshore marine systems, and the biological parameters a community must attain for it to be considered recovered (but see^21,70^). Community recovery is especially difficult to quantify as communities are host to a myriad of biological interactions, and invasive species can insert themselves into those interactions, shaping community structure in unexpected ways via direct and indirect mechanisms^29,45^. Finally, the time required for community recovery after invasive species eradication is uncertain, as is the variability of recovery for different components of the system, requiring a long-term commitment to extensive monitoring.

Here, we move beyond demonstrating increases in native bird abundances after removal of invasive rats, a finding repeatedly shown across multiple studies detailing direct effects of rat eradication on native island species. Our long-term intertidal monitoring data show the changes in densities of rocky intertidal invertebrates and the percentage of intertidal area covered by fleshy algae and aggregating invertebrates after rats were removed from Hawadax Island largely followed the same patterns observed between rat-infested and rat-free islands surveyed in Kurle et al.^12^ indicating a high degree of passive recovery was achieved in a relatively short 11 years. With invasive rats removed, shorebirds have resumed their role as top predator, indirectly shaping the rocky intertidal community (Fig. 2B; see^12^).

More studies focused on understanding and measuring both direct and indirect impacts of invaders, and how communities respond following removal of those impacts, are needed to underscore the widespread conservation successes associated with invasive species eradication, especially on islands. In addition, our data demonstrate that continued eradication of introduced rats from other infested islands in the Aleutian Chain would add to increased bird and rocky intertidal community recoveries within the Alaska Maritime National Wildlife Refuge, thereby contributing to the Refuge’s mission of restoration and protection of natural biodiversity on refuge lands.

## Methods

Hawadax Island (51.80° N, 178.30° E), part of the Alaska Maritime National Wildlife Refuge, is located within the Rat Islands group of the western Aleutian Islands (Fig. 2). The 2,780 ha (6,869 acres) island has steep cliffs along most of the coastline backed by rolling hills and plateaus rising to a small ridge of mountains with a peak elevation of 400 m. There are more than 30 offshore rock stacks and several islets. The largest islet is approximately 4 ha (10 acres) in size and is located 1.7 km off the southeast end of the island (Ayugadak Point). Hawadax Island is a designated Wilderness and has no inhabitants or human facilities.

Rats were introduced to different Aleutian Islands at different times. However, while one island (Little Kiska) became infested in 1990, the rest received rats before 1940 and as early as the 1700′s^42^. Thus, rat effects would currently be largely uniform across islands.

During September and October 2008, the U.S. Fish and Wildlife Service partnered with The Nature Conservancy and Island Conservation to restore seabird breeding habitat on Hawadax Island by removing introduced Norway rats using aerially applied cereal-based bait containing brodifacoum^32^. This was the first aerial rat eradication program conducted in Alaska^32^. Monitoring teams conducted biological surveys from June 1–20, 2008 (pre-eradication), and May 31–June 10, 2013 and June 1–16, 2019 (post-eradication), to coincide with early seasonal breeding activity for most native island bird species.

The protocols used in this study were approved by the Institutional Animal Care and Use Committee (IACUC) of the University of California, Santa Cruz (protocols Crold1004 and Crold1303). The research was performed in strict accordance with the guidelines and regulations in these protocols. Observers did not engage in any contact with the animals while recording their natural behavior and no biological samples were collected.

### Intertidal community

#### Photo plots

We conducted surveys to describe the community structure of intertidal flora and fauna. We used methods similar to those developed by Kurle^71^, who previously conducted studies to document the impacts of Norway rats on marine bird densities and rocky intertidal communities in the Aleutian Islands, including Hawadax Island. In 2008 and 2013, we conducted surveys on eight beaches: five on the north side of the island, and three on the south side. In 2019, we revisited seven of the original eight beaches; due to time constraints, we were unable to survey Beach 6 on the south side of the island (Fig. 2).

**Figure 4:**
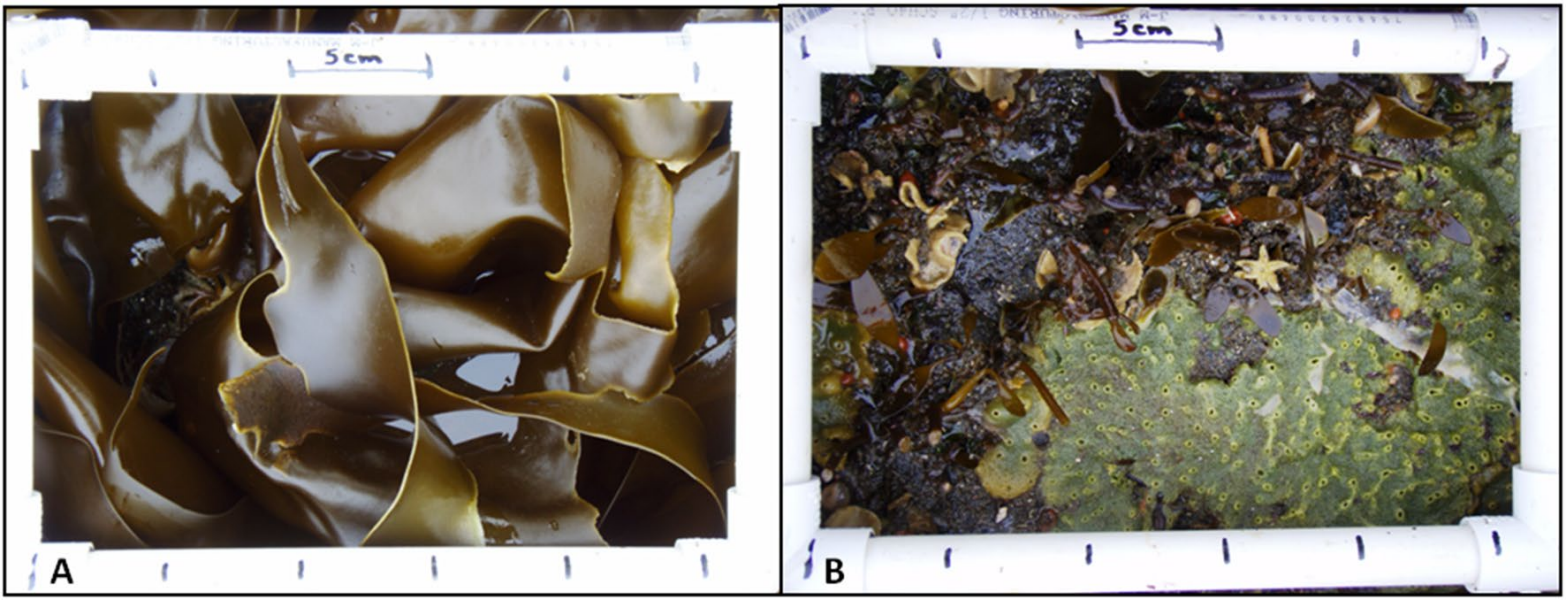
Example of digital photos of intertidal quadrats. A digital photo was taken first of the surface coverage of the plot (**A**). A second photo of the exact plot (**B**) was taken after the algal layer was removed to reveal the under-story community. Photos by J. Curl.

At each beach, we conducted two transects in both the low and mid intertidal zones. The lower intertidal zones were dominated by algae from the genera *Alaria* and *Laminaria*, whereas the mid zones were dominated by algae from the genera *Fucus* and *Halosaccion*. For each 30 m transect, we took digital photos of seven, 365 cm^2^ quadrats, located at 5 m intervals. We took two photos every quadrat: the first of the surface coverage, and a second photo after the overlying algal layer was removed to reveal the understory community (Fig. 4).

Seasonal, logistic, financial, and low-tide constraints all limited our field time, precluding lengthy field identification of invertebrates to species. Our major goal with this limited field time was to get clear and accurate photos of our study plots to best replicate the earlier study of Kurle et al.^12^. As a result, we were not able to confidently identify our metrics to species, but instead relied upon the broader taxonomic categorization. While potentially interesting to have more taxonomic detail, the conclusions in our manuscript based upon the broad taxonomic categories are, we feel, robust.

We analyzed 2008 and 2013 intertidal photos using Adobe Photoshop 6.0. We used Adobe Photoshop Elements 2019 to analyze all digital photos taken in 2019. We followed the same data analysis protocols in 2008, 2013, and 2019. For all years, on each photo, we overlaid a digital 6 × 9 rectangular grid and calculated percent cover as the ratio of the number of each species lying below an intersection of the gridline to the total number of intersections within each grid. We calculated percent cover of sessile organisms (barnacles, sponges, and tunicates). We estimated the percent cover of larger algae (coralline algae and all fleshy algal species) by counting the percent cover of stipes that remained after the removal of the algal blades. We counted the number of individual mobile invertebrates (anemones, snails, limpets, mussels, urchins, and sea stars) in each photo to estimate density. We pooled data by beach for statistical analyses and compared the mean percent cover and mean number of individuals per m^2^ pre- (2008) and post- (2013 and 2019) eradication using t-tests (α = 0.05).

### Beach transects

Shorebirds, such as Black Oystercatchers and Glaucous-winged Gulls, are important predators of intertidal organisms^53–56^. To assess their relative abundance, we conducted beach surveys along the entire length of all accessible beaches (n = 16; Fig. 2) on Hawadax Island. Detailed methods are presented in Croll et al.^33^ and are briefly summarized here. An observer slowly walked along each beach transect during morning hours whenever possible (0700–1100), counting all birds seen or heard between the water’s edge and 50 m inland from the storm tide line. For each species, observers recorded aural and visual detections separately. Observers recorded the time and GPS location at the start and end of each transect. We measured the length of each transect following the contour of the beach between waypoints using ArcGIS 10.7^72^. We conducted four to five replicate surveys of each of the 16 beach transects to minimize effects of variation in time and sampling conditions. We calculated the relative abundances for Black Oystercatchers and Glaucous-winged Gulls by dividing the total count of birds detected per beach transect by the length of the transect (birds km^−1^). We then averaged the five replicate surveys for each beach and used the averaged counts for each beach each year as a sample. We then compared pre- (2008) and post-eradication (2013, 2019) results using nonparametric Van der Waerden tests, *α* = 0.05.

### Assessing status of rocky intertidal community recovery

An island community may be considered recovered if the components are not significantly different from an unperturbed reference island^73^. To assess the degree to which the rocky intertidal on Hawadax Island may have recovered following rat eradication, we compared the percentage change over time for abundances and percent cover values of intertidal organisms and shorebirds on Hawadax Island pre- vs. post-rat eradication to those observed between islands with and without rats surveyed for Kurle et al.^12^.

## Supplementary Information

Supplementary Information
