# Supplementary material for: Indirect effects of invasive rat removal result in recovery of island rocky intertidal community structure
Source: Sci Rep. 2021 Mar 8;11:5395. doi: 10.1038/s41598-021-84342-2 (PMC7940711; doi:10.1038/s41598-021-84342-2)
Supplement: Supplementary file 1 — Supplementary Information [file 41598_2021_84342_MOESM1_ESM.docx]

**Supplemental Material**

**Indirect effects of invasive rat removal result in recovery of island rocky intertidal community structure**

Carolyn M. Kurle, Kelly Zilliacus, Jenna Sparks, Jen Curl, Mila Bock, Stacey Buckelew, Jeff Williams, Heather Renner, Coral Wolf, Jonathan Plissner, Gregg R. Howald, Bernie R. Tershy, Donald A. Croll


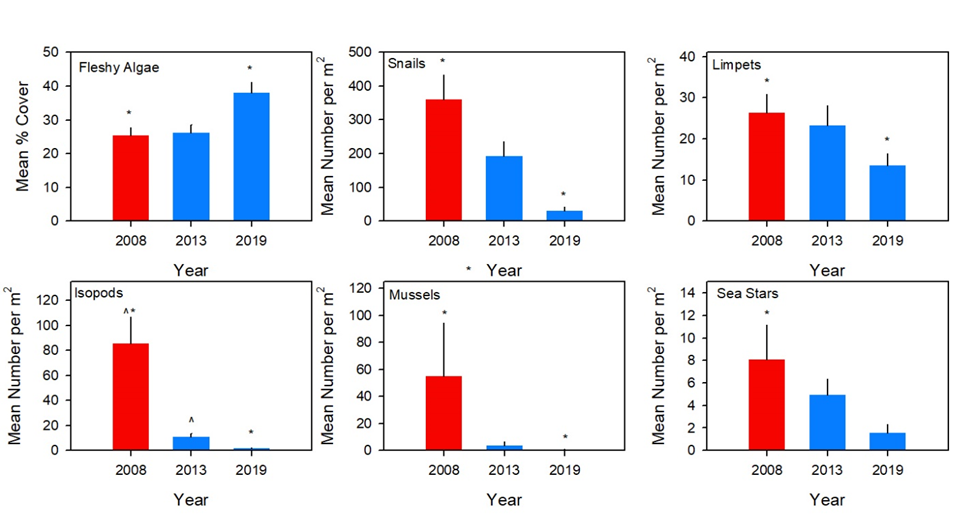
Supplemental Figure 1. Mean ± SE percent cover of fleshy algae and mean ± SE number per m^2^ of snails, limpets, isopods, mussels, and sea stars pre- vs. post-eradication (2008 vs. 2013 and 2019) in intertidal photo plots; ^ indicates significantly different data between 2008 and 2013 whereas * indicates significantly different data between 2008 and 2019, *p* <0.05


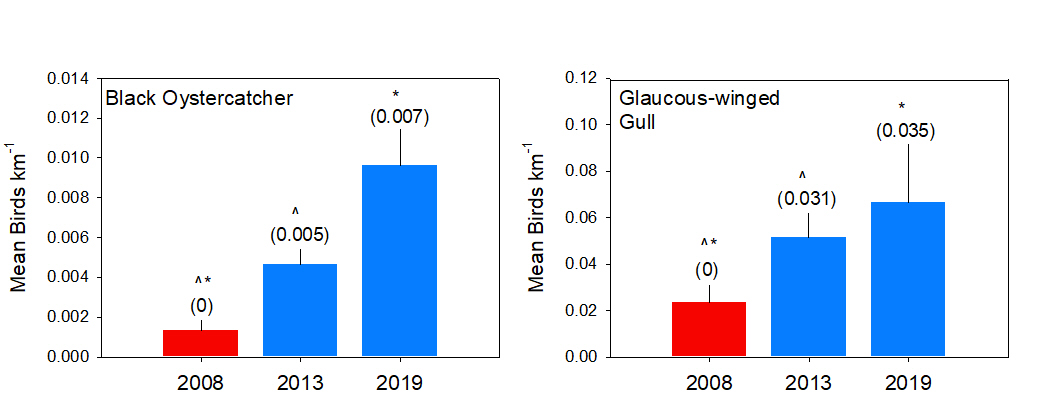


Supplemental Figure 2. Mean ±SE shorebird and seabird abundance on Hawadax Island pre- vs. post-eradication (2008 vs. 2013 and 2019) on beach transects; ^ indicates significantly different data between 2008 and 2013 whereas * indicates significantly different data between 2008 and 2019, *p* <0.05, median values are shown in parentheses.

Supplemental Table 1. The percent change in intertidal organisms and bird species pre (2008) vs. post (2013 and 2019) Norway rat eradication on Hawadax Island, and the percent change in intertidal organisms and birds between islands with and without rats observed in Kurle et al. 2008.

| Species | 2008 to 2013 | 2008 to 2019 | Kurle et al. |
| --- | --- | --- | --- |
| % Encrusting Algae | -73.57 | -87.59 | NA |
| % Fleshy Algae | 2.42 | 49.23 | 67.95 |
| % Geniculate Algae | -19.73 | -29.02 | NA |
| % Barnacles | 9.25 | 15.17 | -81.14 |
| % Sponges | -9.79 | -60.95 | -69.64 |
| % Tunicates | -5.92 | 6.32 | -99.71 |
| Anemones m^-2^ | 13.42 | -74.37 | -69.90 |
| Isopods m^-2^ | -87.54 | -98.02 | NA |
| Limpets m^-2^ | -11.99 | -48.66 | -83.44 |
| Mussels m^-2^ | -93.75 | -98.88 | -96.96 |
| Sea Stars m^-2^ | -38.97 | -80.41 | -97.50 |
| Snails m^-2^ | -46.88 | -91.37 | -82.74 |
| Urchins m^-2^ | -42.71 | -48.62 | NA |
| Black Oystercatcher km^-1^ | 400.00 | 800.00 | 882.75 |
| Glaucous-winged Gull km^-1^ | 126.09 | 191.30 | 913.90 |
